# Rapid Enantiomeric Ratio Determination of Multiple Amino Acids Using Ion Mobility-Mass Spectrometry

**DOI:** 10.3390/molecules30122497

**Published:** 2025-06-06

**Authors:** Wenqing Xu, Estelle Rathahao-Paris, Sandra Alves

**Affiliations:** 1 Sorbonne Université, Faculté des Sciences et de l’Ingénierie, Institut Parisien de Chimie Moléculaire (IPCM), 75005 Paris, France; wenqing.xu@sorbonne-universite.fr; 2 Université Paris-Saclay, CEA, INRAE, Médicaments et Technologies pour la Santé (DMTS), SPI, MetaboHUB, 91191 Gif-sur-Yvette, France

**Keywords:** chiral analysis, enantiomeric ratio, ion mobility-mass spectrometry, flow injection analysis

## Abstract

Chiral analysis is becoming increasingly important across various scientific fields, including chemistry, pharmaceuticals, biosciences, and more recently, metabolomics. In this context, a high-resolution and high-throughput method was developed for the simultaneous determination of the enantiomeric ratio (*er*) of seven pairs of amino acid (AA) enantiomers (Arg, Gln, His, Met, Pro, Tyr, and Trp) using flow injection analysis coupled with ion mobility-mass spectrometry (FIA-IM-MS) technology. Specifically, the Single Ion Mobility Monitoring (SIM^2^) mode on a TIMS-Tof^TM^ instrument enabled the rapid relative quantification of chiral compound mixtures. A linear model accurately described the relationship between enantiomeric ratio and IM-MS response for Arg, Gln, and Pro enantiomers, as evidenced by high R^2^ values and unbiased residuals. In contrast, non-linear trends were observed for His, Tyr, and Trp, where a quadratic model significantly improved the fit. However, the linear model was retained for Met, despite an R^2^ of about 0.98, due to its comparable performance and simplicity. Measurement accuracy was confirmed with very good recovery rates for *er* values of 0.95 and 0.99 across all AAs. Finally, the potential of the FIA-SIM^2^-MS approach in chiral analysis was demonstrated, particularly its ability to provide a reliable and efficient high-throughput tool for accurate *er* determination.

## 1. Introduction

Chirality presents a unique challenge in the life sciences. Many biological molecules, such as amino acids, sugars, and drugs are chiral, and each stereoisomer often exhibits very different effects in biological systems. Chiral amino acids and their metabolites are increasingly being studied for their role in biological processes. L-amino acids (^L^AAs), known as proteogenic, are the primary building blocks of proteins and D-amino acids (^D^AAs). Although they are less abundant in most living organisms, they play critical roles in various biological functions, for example in the central nervous system, where they act as neurotransmitters or neuromodulators. These molecules are not restricted to humans or mammals; they are also present in a wide range of organisms. For example, the peptidoglycan cell walls of bacteria often incorporate D-amino acids alongside their L-counterparts, contributing to structural integrity and resistance to enzymatic degradation. In addition, racemization (the conversion of L-amino acids to their D-forms) can occur naturally over time or be induced by food processing methods such as heating and fermentation, leading to the presence of ^D^AAs in various dietary sources [1,2]. Importantly, disruptions in ^D^AA metabolism have been linked to a variety of pathological conditions. These disturbances are thought to affect key biological processes such as neurotransmission, metabolic regulation, and cell proliferation. As a result, there is growing interest in chiral AA analysis for diagnostic, prognostic, and therapeutic applications [2,3].

Therefore, the analysis of chiral AAs is highly desirable for characterizing individual enantiomers and determining their proportions in a mixture [4]. Since the first experimental observations of chiral molecules, numerous spectroscopic and chromatographic techniques have been developed for this purpose [5]. Among these methods, chiral chromatography is the most commonly used, thanks to the wide availability of chiral stationary phases, selectors, and derivatization agents for various analytes. It offers high sensitivity and resolution, making it particularly effective for enantiomer separation and analysis [6,7]. Alternatively, mass spectrometry (MS) has emerged as a promising technique for chiral analysis, offering advantages in speed, specificity, and sensitivity. However, standalone MS cannot resolve (stereo)isomeric compounds with identical elemental compositions, highlighting the need for advanced MS-based methods [8,9]. Notably, MS methods that typically involve MS/MS experiments, such as Cooks’ kinetic method, have been proposed, allowing enantiomer distinction through the detection of variations (even subtle ones) in stereoselective fragmentation patterns [10,11,12].

Coupling chiral chromatography with mass spectrometry combines the advantages of both techniques, offering the high selectivity of chiral separation with the sensitivity and quantitative capabilities of MS for enhanced chiral analysis. Alternative separation methods, such as supercritical fluid chromatography, capillary electrophoresis, or ion mobility spectrometry, can be also integrated with MS to further improve enantiomer characterization [5]. Such coupling enables rapid analysis while maintaining good separation performance, making it particularly useful for the analysis of complex mixtures, such as those encountered in metabolomics or pharmaceutical research. Ion mobility-mass spectrometry (IM-MS) appears to be a particularly attractive analytical method for (stereo)isomer characterization. However, the IM-MS analysis of isomer compounds remains challenging, with difficulties related to the type of isomerism, i.e., how close their respective mobilities are [13]. Therefore, it often requires high ion mobility resolution conditions, which can be achieved through recent technical developments in ion mobility, such as Trapped Ion Mobility Spectrometry (TIMS), Structures for Lossless Ion Manipulations, and cyclic IM instrumentation [14].

Chiral analysis using the IM-MS approach systematically involves the generation of diastereomer species, by in-solution chemical derivatization [15,16,17,18], gas-phase production of non-covalent assemblies [19,20,21,22,23,24,25], or a combination of these [18]. In situ gas-phase complex formation is fast and easy, requiring no sample preparation or purification. A chiral selector with specific gas-phase behavior toward an enantiomer of interest is typically used, although self-association can also occur [14,26]. The choice of selector is critical and often compound-dependent [15,16,17,18,24]. Note that transition metals are generally used to promote rigid structures that enhance conformational differences among diastereoisomer complexes. The gas-phase formation of multimer ions using various types of chiral selectors has been successfully applied in the characterization of chiral AAs using the IM-MS approach, often as high assembly complexed species that are capable of inducing pronounced conformational differences among diastereomeric species [21]. Previous work has demonstrated the differentiation of chiral AAs as coppered dimeric ions using a TIMS instrument under high-resolution conditions [27], which can be achieved with a small ion mobility detection range [28,29]. Such a targeted approach, termed Single Ion Mobility Monitoring (SIM^2^), has shown potential for distinguishing AA enantiomers [27] and breast milk oligosaccharide isomers [30], but also for quantifying the latter [31].

In this study, the accurate determination of the enantiomeric ratio (*er*) of multiple chiral AAs in mixtures, i.e., Arg, Gln, His, Met, Pro, Trp, and Tyr, was investigated by using SIM^2^ method. The use of IM-MS has been explored previously to determine the *er* of chiral AAs [14,15,16,17,18,19,20,21,22,26]. Notably, Li et al. used a derivatization strategy and a U-shaped ion mobility-mass spectrometer to achieve AA enantiomer detection with relative content below 1% [15,16]. In our study, the feasibility of combining direct introduction by flow injection analysis (FIA) with the high-resolution SIM^2^ mode of the TIMS instrument for rapid and simultaneous *er* determinations was investigated. Although baseline ion mobility separation was not achieved for all seven AA pairs, regression analysis of the obtained seven-level calibration curves showed strong relationships between the *er* values and the responses of each AA enantiomer.

## 2. Results

**Figure 1:**
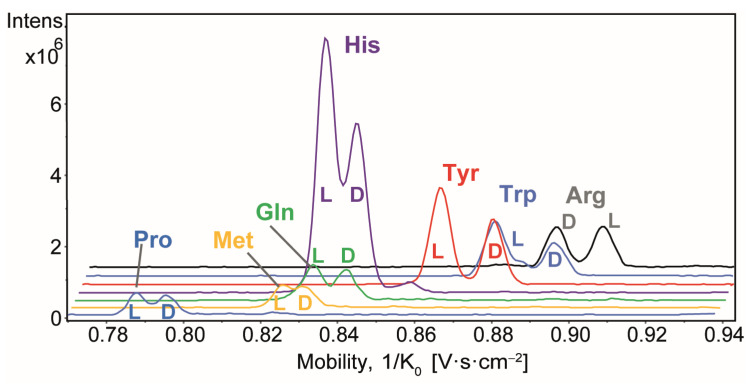
Stacked view of extracted ion mobility spectra of copper dimer [^L+D^AA + ^L^Phe − 2H + CuII]^+^ ions at m/z 342.1, 376.0, 373.1, 382.1, 408.1, 431.1, and 401.1 for the Pro, Met, Gln, His, Tyr, Trp, and Arg enantiomers, respectively, from the FIA-SIM^2^-MS analysis of a racemic AA mixture (*er* = 0.5). Each chiral AA (L and D enantiomers) was at a concentration of 1 µM, the ^L^Phe chiral selector at 10 µM and CuII at 20 µM. Note that the inverse reduced ion mobility (1/K_0_) values of each complex ions are reported in Table S1 of the Supplementary Materials.

The advantage of the SIM^2^ method, which provides high mobility resolution capabilities of the TIMS instrument, has been demonstrated for the chiral analysis of amino acids, in a previous work [27]. In this study, the same method was applied in FIA mode, enabling rapid and high-throughput automated analysis. Under these conditions, all seven pairs of AA enantiomers, i.e., Arg, Gln, His, Met, Pro, Trp, and Tyr, could be detected simultaneously in a single acquisition as copper dimer [^L+D^AA + ^L^Phe − 2H + CuII]^+^ ions, with ^L^Phe acting as a chiral selector. All enantiomers were successfully resolved by ion mobility, as displayed in Figure 1, with peak-to-peak resolution (R_p-p_) values ranging from 0.7 (for Met, which implies about 50% overlap between two ion mobility peaks) to 1.4 (for Trp, which was nearly baseline separated by ion mobility) (see Table S1 in Supplementary Materials) [13]. Nevertheless, the ion mobility peaks were sufficiently resolved to allow further investigation into the relative quantification of all copper-bound dimer species.

However, these complex AA ions did not show the same ionization responses in the IM-MS spectra, despite the same concentrations (1 µM) being analyzed. Higher peak intensities were observed for Arg, His, Trp, and Typ, with the highest intensity noted for His. In contrast, the peak intensities for Gln, Pro, and Met were two to three times lower. For most of the AAs studied, both L- and D-enantiomers exhibited similar abundances, with the exception of His, Trp, and Tyr, which showed notable differences in abundance between the enantiomer ions.

**Figure 2:**
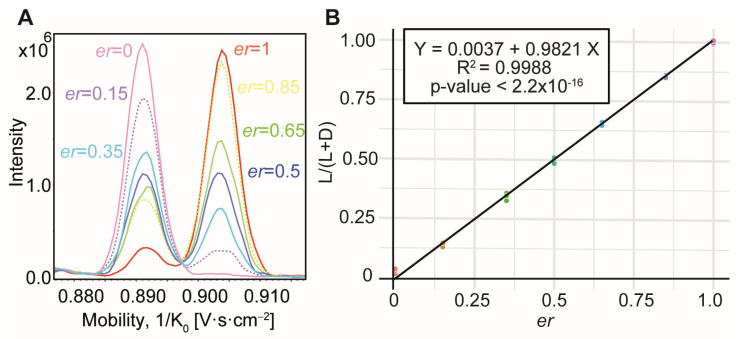
Determination of enantiomeric ratio exemplified for Arg enantiomers: (**A**) extracted ion mobility spectra at m/z 401.1 for the dimer [^L+D^AA + ^L^Phe − 2H + Cu^II^]^+^ ions obtained using FIA-SIM^2^-MS mode, showing the separation of the two Arg enantiomers and the variation of their peak areas as a function of *er* values; (**B**) linear calibration curve obtained by regression analysis, with the 95% confidence interval not visible due to its narrow range.

Taking advantage of the high-throughput capabilities of IM-MS using SIM^2^ mode combined with FIA mode to achieve rapid and accurate chiral analysis, we investigated the use of this method for the simultaneous *er* determination of the seven pairs of chiral AAs (i.e., Arg, Gln, His, Met, Pro, Trp, and Tyr enantiomers). Each *er* level was analyzed in triplicate on each day (*n* = 6 across two days) to ensure reproducibility. Measurements were conducted on two separate days to assess the reproducibility of the ion mobility measurements over time and to ensure quality control in the calibration solution preparation as standard AA mix solutions were freshly prepared. A total of 42 measurements were made for each pair of AA enantiomers. As exemplified in Figure 2A for the Arg enantiomers, the extracted ion mobility spectra of the coppered species showed the signal intensity for both the L- and D-enantiomers as a function of the *er* values, demonstrating the feasibility of our method for *er* determination. Note that at *er* = 1, corresponding to the pure ^L^Arg solution, a minor peak overlapping with the ^D^Arg signal could be observed. This may have been due to a lack of purity in the ^L^Arg solution or the presence of multiple mobility peaks (possibly isomeric adduct ions) corresponding to ^L^Arg. Nevertheless, as shown in Figure 2B, this did not affect the linearity of the response, as the mean calculated *er* value was obtained with a bias below 1% for all repeated measurements.

Regression analysis was performed on all generated data to examine the relationship between the *er* values and the IM-MS measured responses, expressed as L/(L + D) peak area ratios. A linear model provided a good fit for three pairs of AA enantiomers, i.e., Arg, Gln, and Pro, with a high coefficient of determination (R^2^ > 0.99; see Table 1 and Table S4 in Supplementary Materials). The slope coefficients were highly significant (*p*-values < 2.2 × 10^−16^), confirming a strong linear relationship between *er* and the IM-MS responses. For Arg and Pro, the linear models passed near the origin, as the intercepts were not significantly different from zero (*p*-values > 0.05, Table S2). Examination of the residuals showed that, despite their spread, they were randomly scattered around zero, with the median value very close to zero (Table S4) for the three considered pairs of AAs, indicating no significant bias in the model predictions. Such a strong linearity suggested similar ESI ionization efficiencies between D- and L- AA species, as evidenced in Figure 2A.

For His, Tyr, and Trp, the response was not strictly linear (R^2^ < 0.98), especially for His (R^2^ < 0.95, Table 1). In addition, the residual analysis showed a systematic pattern (i.e., a curve) for the linear model for His, Trp, and Tyr (Table S4). This suggests that the relationship between *er* values and the response as the L/(L + D) peak area ratios may not be purely linear. Note that nonlinear relationships between AA enantiomer composition and measured response have been previously reported using CID spectra of protonated trimers [12], where a hyperbolic trend was observed between *er* and the dissociation efficiency, defined as the intensity ratio of protonated dimers to trimers. Also, using the IM-MS approach, a binomial relationship was reported for homo- and hetero-chiral dimer and trimer ions formed by the self-association of several chiral compounds, such as Trp [26]. Here, the quadratic model was tested. It significantly improved the fit to the data for the three AA enantiomers, i.e., His, Tyr, and Trp, as evidenced by the higher R^2^ value (R^2^ > 0.99) and the residual analysis (Table 1 and Table S4). The significant coefficient for the quadratic term (*p*-values < 2.57 × 10^−15^; see Table S2) also highlighted the presence of a non-linear relationship between *er* values and the AA enantiomer response.

In contrast, both linear and quadratic models fit to the Met data well, with similar R^2^ values of about 0.985. However, the quadratic term was statistically insignificant (*p*-value = 0.2795; Table S2), indicating that the quadratic model did not offer a significant improvement over the linear model. Given the comparable fit goodness between the two tested models and the lack of statistical justification of the quadratic model, the linear model was found to be the more appropriate and simpler choice for *er* determination of Met. The lower goodness of linear fit observed for the Met enantiomers, compared to the three AA enantiomer pairs, i.e., Arg, Gln, and Pro, may have been due to the imperfect separation of the two diastereoisomeric complexes (R_p-p_ of 0.7, Table S1) and their relatively low peak intensities (Figure 1). Conversely, the improved fit of the quadratic regression models for the calibration curves of His, Trp, and Tyr evidenced deviations from linearity at high *er* values, corresponding to nearly pure L- or D- enantiomer solutions. This nonlinearity may have been due to saturation effects within the TIMS cell but also the difference in ionization efficiency between the L- or D forms of these AAs. Nevertheless, the quadratic model ensured accurate quantification across the entire *er* range.

A bootstrap resampling procedure with 1.000 resamples was further performed for each pair of AA enantiomers to assess the precision and reliability of the regression model estimates (Table S3). The results indicated that the estimates were robust, with low bias and relatively small standard errors (i.e., R^2^. Mean Squared Error (MSE). Residual Standard Error (RSE) and regression coefficients). An additional ANOVA analysis was performed, and the resulting *p*-value confirmed the adequacy of the model (linear or quadratic) for each pair of AA enantiomers.

To assess the accuracy of our method, FIA-SIM^2^-MS analyses were performed on solutions containing the seven AA enantiomer pairs with known *er* values of 0.95 and 0.99. These *er* values were deliberately chosen to assess the method’s sensitivity in detecting the presence of a substantial excess of one enantiomer with trace amounts of its counterpart—here, a predominance of ^L^AAs with trace levels of ^D^AAs—reflecting conditions typically observed in biological samples. Each solution was analyzed in six replicates on two separate days (*n* = 12). The measured values closely matched the expected values, demonstrating high accuracy, with maximum standard deviations of 0.02 for both *er* = 0.95 and *er* = 0.99 (Table 1). Recovery rates, ranging from 95% to 105%, also confirmed the method’s accuracy, with only minor deviations from the expected *er* values (see Table S2). These differences of ±5% were in line with an acceptable accuracy, typically within ±10% of the theoretical *er* value; see Table S2. The relative standard deviation (RSD) obtained for all chiral AAs was consistently less than 3%, demonstrating the high precision of the measurements and confirming the reliability of the method for determining very low relative proportions of ^D^AA (few %) compared to ^L^AA enantiomers.

In addition, the calibration curves were also evaluated for different AA concentrations. FIA-SIM^2^-MS measurements were performed on two sets of standard solutions at 5 µM, confirming the accuracy and consistency of the results obtained at 1 µM. Almost all calibration curves generated from solutions at 5 µM were very similar to those obtained for 1 µM (see Tables S2, S4 and S5). This suggests that the method provided reliable measurements at both AA concentrations. We, therefore, do not expect any significant bias in the determination of *er* in proteinogenic amino acids using the proposed analytical approach. Although the quadratic term appeared significant for Met (Table S2), no notable change in residual dispersion was observed between the linear and quadratic models (Table S5). Furthermore, a model comparison by ANOVA (see Table S5) supported the use of the linear model for Met at 5 µM. An exception was observed for Pro, where the quadratic model provided a better fit than the linear model at 5 µM, as evidenced by the lower residual dispersion and the statistical significance of the quadratic term (Table S2, S4 and S5), indicating a more accurate representation of the relationship between *er* and the response measured Pro at higher concentrations.

To evaluate the applicability of the method at lower AA concentrations, experiments were also conducted at 0.2 µM concentration of AAs. Among the seven AA enantiomer pairs tested, Pro and Met gave very poor signals. A reliable calibration curve was obtained only for Tyr and Trp, showing R^2^ > 0.99 (*p*-value < 2.2 × 10^−16^) for the quadratic model, which was consistent with results from other concentration levels (Tables S2 and S6). However, for Arg, Gln, and His, the regression models showed poorer fits, with R^2^ values of around 0.97 for Arg and Gln, and a particularly low R^2^ value of 0.76 for His. These results suggest that 0.2 µM is the limit of detection (LOD) for Pro and Met, while their limits of quantification (LOQs) lie between 0.2 µM and 1 µM. For His, the LOD appeared to be slightly below 0.2 µM; however, this concentration is insufficient for accurate *er* determination, suggesting a LOQ greater than 0.2 µM but less than 1 µM. In contrast, Arg, Gln, and especially Tyr and Tyr showed stronger signal intensities and remained detectable and quantifiable at lower concentrations. The LOD and LOQ for Tyr and Trp should both be below 0.2 µM, while the LOQ for Arg and Gln should be around 0.2 µM. These observations were supported by the recovery-based accuracy calculations (Table S2). The recovery ranges were relatively broad for Arg (89–107%) and Gln (94–110%), while Tyr and Trp showed acceptable recovery values (95–103% range) in *er* determination using standard solutions with known *er* (i.e., *er* = 0.95 and *er* = 0.99). Finally, a 0.2 µM concentration certainly represents the lower limit of the working range, which remains considerably lower than that of conventional hyphenated methods. However, it is worth noting that our direct FIA-SIM^2^-MS approach stands out due to its analytical throughput, i.e., less than 3 min per sample, and demonstrated improved sensitivity compared to previous works, involving similar in situ complexation followed by IM-MS. Here, the high-resolution TIMS conditions using SIM^2^ mode enable the ion mobility separation of all copper-bound dimeric ions, resulting in more intense signals (and greater sensitivity) than when using larger complex species [19,21,22].

## 3. Materials and Methods

### 3.1. Materials

All ^L^AAs and ^D^AAs (i.e., arginine (Arg), glutamine (Gln), histidine (His), methionine (Met), Proline (Pro), Phenylalanine (Phe), Tryptophan (Trp), and Tyrosine (Tyr)) and copper(II) chloride (CuCl_2_) were purchased from Sigma-Aldrich (Merck KGaA, Darmstadt, Germany) with purity > 98% for each AA enantiomer. Ultrapure water was produced with a Select HP water purification system (Purite France eau, Lormont, France). Methanol (MeOH) was obtained from VWR Chemicals (Fontenay-sous-Bois, France). The ESI-L low concentration tuning mix (G1969-85000) was purchased from Agilent Technologies (Santa Clara, CA, USA) for the TIMS-tof^TM^ calibration procedure.

### 3.2. Ion Mobility-Mass Spectrometry

First, 5 µL of samples were injected into the TIMS-tof^TM^ mass spectrometer (Bruker Daltonics, Bremen, Germany) in FIA mode at a 20 µL min^−1^ flow rate of methanol/water (1:1. *v*/*v*) using an HPLC autosampler (Elute, Bruker Daltonics, Bremen, Germany).

All IM-MS experiments were conducted using an electrospray ionization (ESI+) source in positive mode, with nitrogen as both spray and drift gas. The source and MS parameters were set as follows: end plate offset, 500 V; capillary voltage, 4000 V; nebulizer gas pressure, 4 psi; dry gas flow, 2 L min^−1^; capillary temperature, 275 C; General tune: deflection 1 delta, 100 V; Funnel 1 RF, 300 V_pp_; Funnel 2 RF, 300 V_pp_; isCID Energy, 0 eV; multipole RF, 500 V_pp_; quadrupole ion energy, 5 eV; low mass, 100 m/z; collision energy, 5 eV. collision RF, 1000 V_pp_. transfer time, 55 µs; and pre pulse storage, 10 µs.

After their production, the ions were separated using the TIMS cell. Unlike classical Drift Tube ion mobility spectrometry [28,29], the TIMS separation principle involves sequential events, including ion accumulation, trapping, and elution. The final elution step offers the possibility to modulate the separation resolution by adjusting the ramp time (in ms) and the ion mobility range, expressed as the inverse reduced mobility (1/K_0_ in $V\cdot s\cdot {cm}^{-2}$) [28]. Under maximum resolution conditions, the SIM^2^ method uses a narrow ion mobility range detection centered on a target ionic species [30]. In the present study, all diastereosiomeric coppered [^L+D^AA + ^L^Phe − H + CuII]^+^ complex ions were resolved using an ion mobility range window from 0.77 to 0.97 $V\cdot s\cdot {cm}^{-2}$ with a 600 ms ramp time (spectral rate of 1.65 Hz); the Ion charge control (ICC) parameter was set at 1.5 Mio. TIMS voltage parameters included: deflection transfer → capillary exit, −55 V; deflection transfer → deflection discard, −50 V; funnel 1 in → deflection transfers, 55 V; accumulation trap → funnel 1 in, 60 V; accumulation exit → accumulation transfers, 0 V; ramp start → accumulation exit, 100 V; funnel 1 RF, 250 V_pp_; and collision cell on, 50 V.

All IM-MS data were acquired in the 100–800 Da mass range. Daily external calibrations in m/z (using quadratic fitting mode) and inverse reduced mobility values (linear mode) were determined using the ESI-L low concentration tuning mix solution. Collision Cross Section (CCS) values were obtained from the measured inverse reduced mobilities (1/K_0_ in $V\cdot s\cdot {cm}^{-2}$) (See Supplemental Materials for additional information about IM-MS parameters).

All operating parameters were controlled through the Hystar 6.2 (for automated FIA injection mode) and oTof control 6.2.8 (for IM-MS experiments). Software and data analyses were performed using DataAnalysis 6.1 (Bruker Daltonics).

### 3.3. Procedure for Enantiomeric Ratio Determination

#### 3.3.1. Preparation of Standard Solutions and Data Collection

Stock solutions of ^L^AAs and ^D^AAs were prepared at a concentration of 10 mM in H_2_O and stored at −20 °C (except for tyrosine (Tyr) at 1 mM). Then, 0.05 mM concentrations of their working solutions were obtained by dilution of their stock solutions. These dilutions where then stored at +4 °C for later use in the preparation of standard solutions for the *er* determination.

Standard solutions were prepared to achieve a 1 µM final concentration of chiral AAs by varying the relative proportions of the ^L^AA and ^D^AA enantiomers. These solutions were prepared in the presence of an excess of CuCl_2_ (20 µM) and ^L^Phe (10 µM) as the chiral selector at seven *er* levels to cover the entire range of *er* values, i.e., from −1 (pure D-enantiomer) to 1 (pure L-enantiomer). The levels included *er* values of 0, 0.15, 0.35, 0.5 (racemic mixture), 0.65, 0.85, and 1. The *er* values were calculated according to the following equation [4]:

er=AALAAL+AAD

where ^L^AA and ^D^AA are the amounts of the L and D- amino acid enantiomers, respectively.

Each standard solution was prepared in two replicates and analyzed over two days (one solution per day) in triplicate (*n* = 6 for each *er* level). This yielded a total of 42 data points, which were used in the regression analysis for the *er*.

Note that to test our approach at higher and lower AA concentrations, two other series of the seven standard solutions, i.e., with the same *er* values, were also prepared at 5 µM and 0.2 µM of final concentration of chiral AAs.

From the generated ion mobility mass spectra for each diastereoisomer [^L+D^AA + ^L^Phe − 2H + Cu^II^]^+^ ions, the areas under the peaks corresponding to the ^L^AA and ^D^AA species of each replicate were integrated and the L/(L + D) ratios were plotted against *er* values.

#### 3.3.2. Regression Analysis

Regression analysis (linear and non-linear fitting) was performed to obtain the best-fit line or curve for the 42 data points for each pair of AA enantiomers. This was done by analyzing the relationship between the *er* values and the corresponding peak area ratios of each enantiomer (i.e., L/(L + D)).

Data analysis was carried out using the R programming language (version 4.4.3) for statistical modeling, with the *lm()* function to create regression models. The *boot()* function from the boot package was used for the bootstrap resampling procedure. The goodness of fit for both linear and quadratic regression models was assessed based on the R^2^ coefficient of determination (or multiple R-squared). Residual plots were also examined to verify the fit of the model to the data. For both models, the 95% confidence intervals were calculated to quantify the uncertainty in the parameter estimates. A bootstrap resampling with 1.000 replacements was also employed.

## 4. Conclusions

This study highlighted the effectiveness of the SIM^2^ method combined with the FIA mode for the rapid and high-resolution chiral analysis of AA enantiomers. All seven AA enantiomer pairs were successfully detected and separated as coppered dimer ions with ^L^Phe as a chiral selector. Although different signal intensities were observed among the chiral AAs studied, with Arg, His, Trp, and Tyr showing stronger responses, the enantiomeric ratio was successfully determined for all AAs using seven-level, six-replicate calibration curves. The linear model provided an accurate and reliable approach for determining enantiomeric ratio for most AAs, while a quadratic model improved data fit for His, Tyr, and Trp. Our method demonstrated high accuracy, confirmed by excellent recovery rates for *er*, with values of 0.95 and 0.99 for all AAs.

The proposed approach for chiral analysis has proven its potential, particularly by providing a reliable and efficient high-throughput method for the accurate determination of enantiomeric ratio. This offers great promise for advancing research in metabolomics and other related areas. Note that although the detection limit of the FIA-SIM^2^−MS method appears higher than several conventional LC−MS methods, its high throughput capability is a valuable advantage. Nevertheless, the integration of an additional separation dimension, such as LC-IM-MS under traditional achiral conditions, may further enhance the applicability of the method and extend its working range.

## Figures and Tables

**Table 1:** Regression output from the data generated by the analysis of standard solutions containing 1 µM chiral AA mixtures with replicated intra-day and inter-day injections. Reproducibility was assessed with repeated measurements from solutions of chiral AA mixes at *er* = 0.95 and *er* = 0.99. The mean calculated *er* values are reported below. Note that the best fit model is indicated in bold.

Chiral	Calibration Curves ^(a)^			Mean Calculated *er* and Standard Deviations ^(b)^			
AA	Equation ^(c)^	R^2^	%RSD	Theoretical *er* of 0.95		Theoretical *er* of 0.99	
Arg	**Y = 0.0037 + 0.9821 X**	**0.9988**	**2.3**	**0.95**	**± 0.01**	**0.99**	**± 0.01**
	Y = 0.0111 + 0.9283 X + 0.0538 X^2^	0.9991	2.0	0.95	± 0.01	0.98	± 0.01
Gln	**Y = −0.023 + 0.9566X**	**0.9949**	**5.1**	**0.95**	**± 0.02**	**1.01**	**± 0.02**
	Y = −0.0015 + 0.8003 X + 0.1563 X^2^	0.9971	3.8	0.94	± 0.02	0.98	± 0.01
His	Y = 0.0244 + 0.8744 X	0.9445	15.1	0.98	± 0.02	1.06	± 0.01
	**Y = 0.1164 + 0.2239 X + 0.6525 X^2^**	**0.9914**	**6.0**	**0.93**	**± 0.01**	**0.97**	**± 0.01**
Met	**Y = 0.0221 + 0.9777 X**	**0.9853**	**7.6**	**0.95**	**± 0.01**	**0.99**	**± 0.01**
	Y = 0.0328 + 0.8978 X + 0.0802 X^2^	0.9859	7.5	0.95	± 0.01	0.98	± 0.01
Pro	**Y = 0.0031 + 0.9788 X**	**0.9952**	**4.7**	**0.95**	**± 0.01**	**1.00**	**± 0.01**
	Y = 0.0187 + 0.8656 X + 0.1132 X^2^	0.9963	4.1	0.94	± 0.01	0.99	± 0.01
Trp	Y = −0.0557 + 0.9696 X	0.9738	12.6	1.01	± 0.01	1.06	± 0.01
	**Y = 0.0161 + 0.4467 X + 0.5229 X^2^**	**0.9978**	**3.7**	**0.96**	**± 0.01**	**0.99**	**± 0.003**
Tyr	Y = −0.0497 + 0.9635 X	0.9760	11.9	1.00	± 0.01	1.06	± 0.002
	**Y = 0.0199 + 0.456 X + 0.5075 X^2^**	**0.9989**	**2.6**	**0.95**	**± 0.01**	**0.99**	**± 0.001**

^(a)^ Six measurements at each *er* levels for all AAs. except His with five measurements at each *er* level. ^(b)^ mean calculated *er* values from 10–12 repeated measurements (for comparison, recovery-based accuracy and precision values are reported in Table S2 in the Supplementary Materials). ^(c)^ *p*-values < 2 × 10^−16^ linear (slope) coefficients. *p*-values < 2.57 × 10^−15^ for the coefficient of the quadratic term (a coefficient in Table S2) except for Arg (*p*-value = 0.0020), Gln (2.49 × 10^−6^), Pro (0.0013), and Met (*p*-value = 0.2795).

## Data Availability

Data are available from the corresponding author upon reasonable request.

## Supplementary Materials

The following supporting information can be downloaded at: https://www.mdpi.com/article/10.3390/molecules30122497/s1. Table S1: Mean values of 1/K0, ΔCCS% and Rp-p measurements for all tested chiral AA at 1 µM concentration. Table S2: Regression analysis results based on FIA-SIM2-MS data for chiral AA standard solutions at different concentrations. Recovery-based accuracy and precision were calculated from repeated FIA-IM-measurements of chiral AA standard solutions with known er values of 0.95 and 0.99. Table S3: Bootstrap results based on data from the analysis of chiral AA standard solutions at 1 µM and 5 µM concentrations with replicated intra-day and inter-day injections (n = 6). Table S4: Results of regression analysis based on the data from the FIA-SIM2-MS analysis of chiral AA standard solutions at 1 µM concentration. Table S5: Results of regression analysis based on the data from the FIA-SIM2-MS analysis of AA standard solutions at 5 µM concentration. Table S6: Results of the data from the FIA-SIM2-MS analysis of chiral AA standard solutions at 0.2 µM concentration.
