# Supplementary material for: Rapid Enantiomeric Ratio Determination of Multiple Amino Acids Using Ion Mobility-Mass Spectrometry
Source: Molecules. 2025 Jun 6;30(12):2497. doi: 10.3390/molecules30122497 (PMC12195981; doi:10.3390/molecules30122497)
Supplement: Supplementary file 1 [file molecules-30-02497-s001.zip › molecules-3632519-supplementary.pdf]

Supporting information

Rapid Enantiomeric Ratio Determination of Multiple Amino Acids Using Ion Mobility-Mass Spectrometry

Wenqing Xu [1], Estelle Rathahao-Paris [1,2,*] and Sandra Alves [1,*]

[1] Sorbonne Université, Faculté des Sciences et de l'Ingénierie, Institut Parisien de Chimie Moléculaire (IPCM), 75005 Paris, France
[2] Université Paris-Saclay, CEA, INRAE, Médicaments et Technologies pour la Santé (DMTS), SPI, MetaboHUB, 91191 Gif-sur-Yvette, France
* Correspondence: estelle.paris@inrae.fr (E.R.-P.); sandra.alves@sorbonne-universite.fr (S.A.)

Ion mobility-mass spectrometry

The separation capacity of the Ion mobility can be assessed using the peak resolution (R) determined from the measured extracted ion mobility signals ($1/K_0$ the inverse reduced mobility *versus* intensity) according $R = K_0/\Delta K_0$ where K_0 is the reduced mobility of the respective analyzed diastereomer and ΔK_0 its peak width at half maximum (FWHM).

Alternatively, the ΔCCS% value can be used to quantify the IM separation capacity, it is defined as the CCS difference between the two peaks of L-AA and D-AA divided by the average of their CCS values:

$$\Delta CCS\% = 2\left(\frac{CCS_L - CCS_D}{CCS_L + CCS_D}\right) \times 100$$

By analogy with chromatographic separation, a peak-to-peak resolution ($R_{p\text{-}p}$) can also be calculated using the following equation:

$$R_{p-p} = 1.18\frac{1/K_{0,L} - 1/K_{0,D}}{\Delta(1/K_{0,L}) - \Delta(1/K_{0,D})}$$

where $1/K_{0,D}$ *and* $1/K_{0,L}$ refer to the inverse reduced ion mobility of the D- and L-enantiomers, respectively (detected as diastereomeric dimer adduct ions), and $\Delta(1/K_{0,D})$ *and* $\Delta(1/K_{0,L})$ correspond to their respective peak widths measured at half maximum height (i.e. full width at half maximum). A coefficient of 1.18 (2.355σ/2) is included to express peak widths in terms of half-height.

Of course, a higher ΔCCS value corresponds to a higher $R_{p\text{-}p}$, but $R_{p\text{-}p}$ also takes into account the peak width (and thus ion mobility peak resolution) which may vary according to the AA ion mobility signal, for example, due to unresolved ion structures.

Typically, an $R_{p\text{-}p}$ value of 0.6 corresponds to approximately 50% overlap between two peaks (half-height separation), $R_{p\text{-}p}$ = 1.0 represents about 10% overlap, and $R_{p\text{-}p} \geq 2.0$ indicates baseline-separated peaks with no overlap (See **Table S1**).

Table S1. Mean values of $1/K_0$, ∆CCS% and $R_{p\text{-}p}$ measurements for all tested chiral AA at 1 µM concentration. They were averaged from replicate intraday and interday injections (n=6), for a total of 42 measurements for each studied chiral AA.

AA	Studied Species	m/z		$1/K_{0,L}$		$1/K_{0,D}$		∆CCS%	$\lvert R_{pp} \rvert$
**Arg**	[**Arg**+^{L}Phe-H+Cu]$^+$	401.1091	± 0.0012	0,9215	± 0,0152	0,9094	± 0,0147	1,3%	1,1
**Gln**	[**Gln** +^{L}Phe-H+Cu]$^+$	373.0690	± 0.0204	0,8472	± 0,0141	0,8575	± 0,0140	-1,2%	1,0
**His**	[**His**+^{L}Phe-H+Cu]$^+$	382.0669	± 0.0012	0,8493	± 0,0160	0,8571	±0,0167	-0,9%	0,9
**Met**	[**Met**+^{L}Phe-H+Cu]$^+$	376.0303	± 0.1340	0,8413	± 0,0138	0,8470	± 0,0136	-0,7%	0,7
**Pro**	[**Pro**+^{L}Phe-H+Cu]$^+$	342.0609	± 0.0013	0,8040	± 0,0134	0,8119	± 0,0134	-0,9%	0,8
**Trp**	[**Trp**+^{L}Phe-H+Cu]$^+$	431.0871	± 0.0010	0,8947	± 0,0148	0,9101	± 0,0150	-1,7%	1,4
**Tyr**	[**Tyr**+^{L}Phe-H+Cu]$^+$	408.0712	± 0.0012	0,8816	± 0,0143	0,8944	± 0,0147	-1,4%	1,2

Table S2. Regression analysis results based on FIA-SIM²-MS data for chiral AA standard solutions at different concentrations. Recovery-based accuracy and precision were calculated from repeated FIA-IM-measurements of chiral AA standard solutions with known *er* values of +0.95 and +0.99. Note that linear regression follows the model Y = c + b X, and quadratic regression follows Y = c + b X + a X^2.

AA	[AA]	Calibration curves*							Recovery accuracy & Precision (in RSD%) **	
		Equation	R^2	%RSD	RSE	*p-value* of regression coefficients*** a	b	c	*er*=0.95	*er*=0.99
**Arg**	**0.2 µM**	**Y = 0.1856 + 0.7074 X**	**0.9675**	**8.3**	**0.0458**	**-**	**1.35 x10^{-15}**	**3.00 x10^{-9}**	**89-107% (7.2)**	**89-106% (7.3)**
		Y = 0.1919 + 0.6614 X + 0.046 X^2	0.9678	8.2	0.0468	0.665	9.64x10^{-6}	1.58 x10^{-7}	89-106% (6.8)	89-105% (6.9)
	**1 µM**	**Y = 0.0037 + 0.9821 X**	**0.9988**	**2.3**	**0.0115**	**-**	**< 2x10^{-16}**	**0.251**	**99-101% (0.7)**	**99-101% (0.7)**
		Y = 0.0111 + 0.9283 X + 0.0538 X^2	0.9991	2.0	0.0103	0.00203	< 2x10^{-16}	0.0040	99-100% (0.6)	98-100% (0.7)
	**5 µM**	**Y = 0.0032 + 0.9762 X**	**0.9995**	**1.4**	**0.0071**	**-**	**< 2x10^{-16}**	**0.1250**	**100-101% (0.2)**	**100-102% (0.5)**
		Y = 0.0113 + 0.9203 X + 0.0549 X^2	0.9998	0.9	0.0047	1.87x10^{-8}	< 2x10^{-16}	1.72x10^{-7}	100-101% (0.2)	100-101% (0.5)
**Gln**	**0.2 µM**	**Y = 0.0234 + 0.8292 X**	**0.9717**	**11.1**	**0.0500**	**-**	**3.64 x10^{-16}**	**0.2460**	**94-106% (4.6)**	**95-110% (5.9)**
		Y = 0.0581 + 0.5768 X + 0.2524 X^2	0.9793	9.5	0.0439	0.0192	2.35x10^{-5}	0.0160	93-102% (3.7)	93-104% (4.7)
	**1 µM**	**Y = -0.023 + 0.9566 X**	**0.9949**	**5.1**	**0.0237**	**-**	**< 2x10^{-16}**	**0.0011**	**95-105% (2.8)**	**99-105% (1.7)**
		Y = -0.0015 + 0.8003 X + 0.1563 X^2	0.9971	3.8	0.0180	2.49x10^{-6}	< 2x10^{-16}	0.8080	94-102% (2.5)	97-102% (1.5)
	**5 µM**	**Y = -0.0211 + 0.9434 X**	**0.9947**	**5.1**	**0.0236**	**-**	**< 2x10^{-16}**	**0.0037**	**96-104 % (3.0)**	**94-105 % (3.2)**
		Y = 0.0021 + 0.7835 X + 0.1571 X^2	0.9970	3.8	0.0180	5.18x10^{-6}	< 2x10^{-16}	0.756	95-102 % (2.7)	95-103 % (2.8)
**His**	**0.2 µM**	Y = 0.2138 + 0.5017 X	0.5296	35.0	0.1670	-	0.0002	0.0040	79 168% (32.1)	165-189% (5.7)
		Y = 0.3711 - 0.645 X + 1.1467 X^2	0.7639	24.8	0.1215	0.0005	0.0349	8.4x10^{-6}	90-103% (5.9)	100-103% (1.3)
	**1 µM**	Y = 0.0244 + 0.8744 X	0.9445	15.1	0.0714	-	<2x10^{-16}	0.265	99-107 % (2.3)	105-109 % (0.9%)
		**Y = 0.1164 + 0.2239 X + 0.6525 X^2**	**0.9914**	**6.0**	**0.0285**	**2.57x10^{-15}**	**7.84x10^{-5}**	**2.35x10^{-12}**	**95-99 % (1.5)**	**97-99 % (0.6 %)**
	**5 µM**	Y = -0.0386 + 0.9908 X	0.9853	8.8	0.0415	-	< 2x10^{-16}	0.0026	90-106 % (4.1)	103-105 % (0.6 %)
		**Y = 0.0141 + 0.6275 X + 0.3568 X^2**	**0.9959**	**4.6**	**0.0221**	**6.62x10^{-12}**	**< 2x10^{-16}**	**0.0982**	**90-102 % (3.3)**	**99-100 % (0.5 %)**
**Met**	**0.2 µM**	not quantifiable								
	**1 µM**	**Y = 0.0221 + 0.9777 X**	**0.9853**	**7.6**	**0.0417**	**-**	**<2 x10^{-16}**	**0.116**	**99-100 % (0.9)**	**97-100 % (0.8)**
		Y = 0.0328 + 0.8978 X + 0.0802 X^2	0.9859	7.5	0.0415	0.2795	1.22 x10^{-12}	0.0592	98-101 % (0.9)	97-99 % (0.8)
	**5 µM**	**Y = -0.0403 + 1.003 X**	**0.9771**	**11.1**	**0.0526**	**-**	**<2x10^{-16}**	**0.0115**	**100-105 % (1.5)**	**101-104 % (0.9)**
		Y = 0.006 + 0.6839 X + 0.3133 X^2	0.9851	8.9	0.0431	7.85 x10^{-5}	4.82 x10^{-11}	0.712	98-102 % (1.2)	98-100 % (0.7)

* 5 measurements for His; 6 for all other studied AA enantiomer pairs at 1 µM and 5 µM; 3 measurements for 0.2 µM

** Recovery-based accuracy reflects how closely repeated *er* measurements (n=10-12 for 1 µM and 5 µM and n=6 for 0.2 µM) n = 12) align with the true values of 0.95 and 0.99, (with 100% representing ideally accuracy), while the precision is expressed as the relative standard deviation (RSD in %) and indicates the consistency the repeated measurements.

*** F-statistics were calculated with 2 and 37 degrees of freedom for numerator and denominator, respectively.

RSE: Residual Standard Error (or Residual Standard Deviation)

RSD: Relative Standard Deviation (in %)

Note that the models pass near the origin when the intercepts are not significantly different from zero (p-values > 0.05)

Table S2 (continued). Regression analysis results based on FIA-SIM²-MS data for chiral AA standard solutions at different concentrations. Recovery-based accuracy and precision were calculated from repeated FIA-IM-measurements of chiral AA standard solutions with known *er* values of +0.95 and +0.99. Note that linear regression follows the model Y = c + b X, and quadratic regression follows Y = c + b X + a X^2.

AA	[AA]	Calibration curves*							Recovery accuracy & Precision (% RSD) **	
		Equation	R^2	%RSD	RSE	*p-value* of regression coefficients***			*er*=0.95	*er*=0.99
						a	b	c		
Pro	0.2 µM	not quantifiable								
	1 µM	**Y = 0.0031 + 0.9788 X**	**0.9952**	**4.7**	**0.0234**	**-**	**$<2x10^{-16}$**	**0.629**	**97-102 % (1.4)**	**100-102 % (0.8)**
		Y = 0.0187 + 0.8656 X + 0.1132 X^2	0.9963	4.1	0.0207	0.0013	$<2x10^{-16}$	0.0141	96-101 % (1.3)	98-100 % (0.7)
	5 µM	Y = -0.0353 + 0.9829 X	0.9845	9.0	0.0423		$< 2 x10^{-16}$	0.0062	100-104 % (1.3)	103-105 % (0.6)
		**Y = 0.0175 + 0.6187 X + 0.3576 X^2**	**0.9954**	**4.9**	**0.0234**	**$3.09 x10^{-11}$**	**$< 2x10^{-16}$**	**0.0549**	**98-101 % (1.0)**	**99-101 % (0.4)**
Trp	0.2 µM	Y = -0.038 + 0.9241 X	0.9676	13.7	0.0597	-	$1.31 x10^{-15}$	0.121	98-108% (3.4)	100-106 % (2.4)
		**Y = 0.0347 + 0.3939 X + 0.5302 X^2**	**0.9945**	**5.6**	**0.0252**	**$2.21 x10^{-8}$**	**$2.66 x10^{-6}$**	**0.0126**	**95-102 % (2.4)**	**95-99 % (1.7)**
	1 µM	Y = -0.0557 + 0.9696 X	0.9738	12.6	0.0548	-	$< 2x10^{-16}$	0.0007	105-107 % (0.8)	106-108 % (0.5)
		**Y = 0.0161 + 0.4467 X + 0.5229 X^2**	**0.9978**	**3.7**	**0.0162**	**$< 2x10^{-16}$**	**$< 2x10^{-16}$**	**0.00765**	**100-101 % (0.6)**	**100-101 % (0.3)**
	5 µM	Y = -0.079 + 0.984 X	0.9674	14.5	0.0619	-	$<2x10^{-16}$	$7.72 x10^{-5}$	105-107 % (0.7)	108-109 % (0.2)
		**Y = 0.012 + 0.3562 X + 0.6164 X^2**	**0.9991**	**2.4**	**0.0106**	**$<2x10^{-16}$**	**$<2x10^{-16}$**	**0.0045**	**100-101 % (0.5)**	**100-101 % (0.1)**
Tyr	0.2 µM	Y = -0.0387 + 0.932 X	0.9760	11.8	0.0516	-	$<2x10^{-16}$	0.0704	96-100 % (1.6)	96-100 % (1.4)
		**Y = 0.0288 + 0.44 X + 0.492 X^2**	**0.9991**	**2.3**	**0.0105**	**$4.47 x10^{-14}$**	**$6.14 x10^{-13}$**	**$3.22 x10^{-5}$**	**100-103 % (1.1)**	**99-101 % (0.9)**
	1 µM	Y = -0.0497 + 0.9635 X	0.976	11.9	0.0521	-	$<2x10^{-16}$	0.0013	104-107 % (0.8)	107-108 % (0.2)
		**Y = 0.0199 + 0.456 X + 0.5075 X^2**	**0.9989**	**2.6**	**0.0114**	**$< 2x10^{-16}$**	**$<2x10^{-16}$**	**$1.33x10^{-5}$**	**100-101 % (0.5)**	**100-101 % (0.1)**
	5 µM	Y = -0.0593 + 0.9739 X	0.9757	11.9	0.0526		$<2x10^{-16}$	0.0004	104-105 % (0.3)	107-108 % (0.4)
		**Y = 0.0166 + 0.4506 X + 0.5137 X^2**	**0.9984**	**3.1**	**0.0137**	**$<2x10^{-16}$**	**$<2x10^{-16}$**	**0.00268**	**100-100 % (0.2)**	**100-101 % (0.3)**

* 5 measurements for His; 6 for all other studied AA enantiomer pairs at 1 µM and 5 µM; 3 measurements for 0.2 µM

** Recovery-based accuracy reflects how closely repeated *er* measurements (n=10-12 for 1 µM and 5 µM and n=6 for 0.2 µM) n = 12) align with the true values of 0.95 and 0.99, (with 100% representing ideally accuracy), while the precision is expressed as the relative standard deviation (RSD in %) and indicates the consistency the repeated measurements.

*** F-statistics were calculated with 2 and 37 degrees of freedom for numerator and denominator, respectively.

RSE: Residual Standard Error (or Residual Standard Deviation)

RSD: Relative Standard Deviation (in %)

Note that the models pass near the origin when the intercepts are not significantly different from zero (p-values > 0.05)

Table S3. Bootstrap results based on data from the analysis of chiral AA standard solutions at 1 µM and 5 µM concentrations with replicated intra-day and inter-day injections (n= 6). Note that linear regression follows the model Y = c + b X, and quadratic regression follows Y = c + b X + a X^2. Linear and quadratic models were tested and compared by ANOVA.

AA (con.)	Model	Model comparison: linear *vs.* quadratic (p-value)	R^2	MSE	RSE	Coeff. c (p-value)	Coeff. b (p-value)	Coeff. a (p-value)
**Arg (1 µM)**	**linear**	0.7266	0.9994	$6.57x10^{-5}$	0.0081	0.0026 (0.7017)	0.98706 ($2.96x10^{-9}$)	-
	**quadratic**		0.9994	$6.35x10^{-5}$	0.0080	0.0047 (0.6292)	0.9718 ($2.16x10^{-5}$)	0.0153 (0.7266)
**Arg (5 µM)**	**linear**	0.0438	0,9997	$2.87x10^{-5}$	0.0054	0.0007 (0.8712)	0.9771 ($3.94\ x10^{-10}$)	-
	**quadratic**		0,9999	$9.23\ x10^{-6}$	0.0030	0.0069 (0.1157)	0.9319 ($5.45x10^{-7}$)	0.0452 (0.0438)
**Gln (1 µM)**	**linear**	0.0220	0.9911	0.0010	0.0310	-0.0344 (0.2251)	0.9729 ($2.55x10^{-6}$)	-
	**quadratic**		0.9979	0.0002	0.0149	0.0037 (0.8370)	0.6951 (0.0009)	0.2778 (0.0220)
**Gln (5 µM)**	**linear**	0.1561	0.9898	0.0011	0.0324	-0.0294 (0.3101)	0.9524 ($3.56x10^{-6}$)	-
	**quadratic**		0.9942	0.0006	0.0245	0.0005 (0,9854)	0.7342 (0.0049)	0.2182 (0.1561)
**His (1 µM)**	**linear**	0.0049	0.9629	0.0034	0.0584	0.02918 (0.5604)	0.8853 ($9.13x10^{-5}$)	-
	**quadratic**		0.9953	0.0004	0.0196	0.1064 (0.0088)	0,3225 (0.0365)	0.5628 (0.0049)
**His (5 µM)**	**linear**	0.0461	0.9845	0.0017	0.0414	-0,0436 (0.2458)	0.9831 ($1.01x10^{-5}$)	-
	**quadratic**		0.9949	0.0006	0.0237	0.0039 (0.8911)	0,6363 (0.0073)	0.3468 (0.0461)
**Met (1 µM)**	**linear**	0.9901	0.9893	0.0012	0.0353	-0.0007 (0.9823)	1.0108 ($4.04x10^{-6}$)	-
	**quadratic**		0.9893	0.0013	0.0353	-0.0010 (0.9816)	1.0132 (0.0058)	-0.0024 (0.9901)
**Met (5 µM)**	**linear**	0.3314	0.9676	0.0037	0.0610	-0.0285 (0.5856)	0.9916 ($6.49x10^{-5}$)	-
	**quadratic**		0.9752	0.0028	0.0534	0.0129 (0.8425)	0.6901 (0.0722)	0.3015 (0.3314)

MSE: Mean Squared Error,

RSE: Residual Standard Error (or Residual Standard Deviation)

Table S3 (continued). Bootstrap results based on data from the analysis of chiral AA standard solutions at 1 µM and 5 µM concentrations with replicated intra-day and inter-day injections (n= 6). Note that linear regression follows the model Y = c + b X, and quadratic regression follows $Y = c + b X + a X^2$. Linear and quadratic models were tested and compared by ANOVA.

AA (con.)	Model	Model comparison: linear *vs.* quadratic (p-value)	R^2	MSE	RSE	Coeff. c (p-value)	Coeff. b (p-value)	Coeff. a (p-value)
Pro (1 µM)	linear	0.4088	0.9967	0.0003	0.0188	0.0031 (0.8444)	0.9754 ($2.09 x 10^{-7}$)	-
	quadratic		0.9973	0.0003	0.0171	0.0141 (0.5067)	0.8949 (0.0006)	0.0804 (0.4088)
Pro (5 µM)	linear	0.0288	0.9815	0.0020	0.0448	-0.0342 (0.3838)	0.9713 ($1.58 x 10^{-5}$)	-
	quadratic		0.9951	0.0005	0.0230	0.0196 (0.4945)	0.5784 (0.0092)	0.3929 (0.0288)
Trp (1 µM)	linear	0.0010	0.9758	0.0026	0.0510	-0.0466 (0.3067)	0.9649 ($3.12 x 10^{-5}$)	-
	quadratic		0.9988	0.0001	0.0115	0.0232 (0,1517)	0.4562 (0,0017)	0.5087 (0,0010)
Trp (5 µM)	linear	0.0001	0.9672	0.0036	0.0604	-0.0765 (0.1751)	0.9761 ($6.69 x 10^{-5}$)	-
	quadratic		0.9994	$7.19 x 10^{-5}$	0,0085	0.0074 (0.4855)	0.3644 (0,0013)	0.6117 (0.0001)
Tyr (1 µM)	linear	0.0010	0.9765	0.0025	0.0503	-0.0478 (0.2893)	0.9645 ($2.90 x 10^{-5}$)	-
	quadratic		0.9988	0.0001	0.0113	0.0210 (0.1785)	0.4633 (0.0015)	0.5012 (0.0010)
Tyr (5 µM)	linear	0.0028	0.9763	0.0026	0.0505	-0.0553 (0.2303)	0.9659 ($2.96 x 10^{-5}$)	-
	quadratic		0.9980	0.0002	0.0147	0.0125 (0.4984)	0.4714 (0.0039)	0.4946 (0.0028)

MSE: Mean Squared Error,
RSE: Residual Standard Error (or Residual Standard Deviation)

Table S4. Results of regression analysis based on the data from the FIA-SIM²-MS analysis of chiral AA standard solutions at 1 µM concentration. All calibration curves are showed with 95% confidence intervals (which are not visible for some curves due to their narrow range).

	**Linear model**	**Quadratic model**
**Arg**	Y = 0.0037 + 0.9821 X<br>R^2=0.9988, p-value < $2.2x10^{-16}$	Y = 0.0111 + 0.9283 X + 0.0538 X^2<br>R^2=0.9991, p-value < $2.2x10^{-16}$
	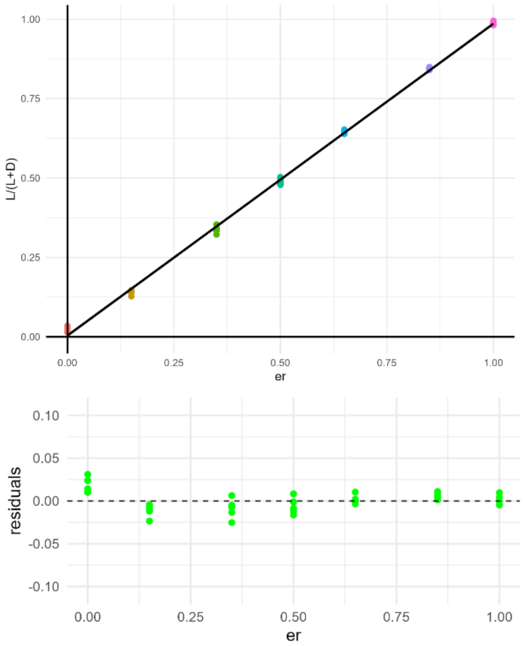	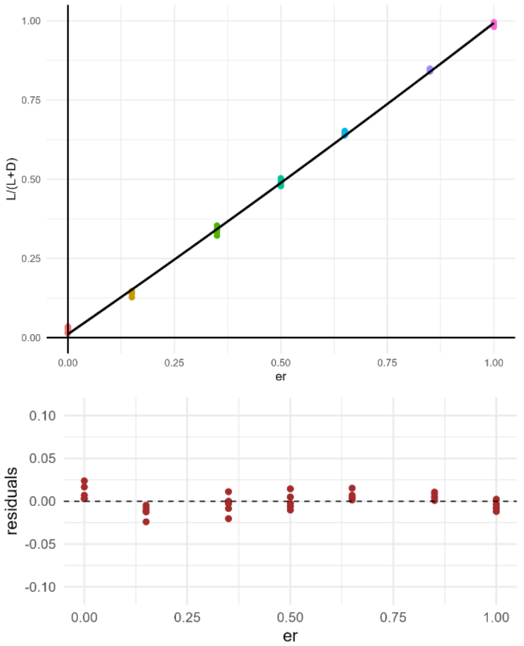
**Gln**	Y = - 0.023 + 0.9566 X<br>R^2=0.9949, p-value < $2.2x10^{-16}$<br>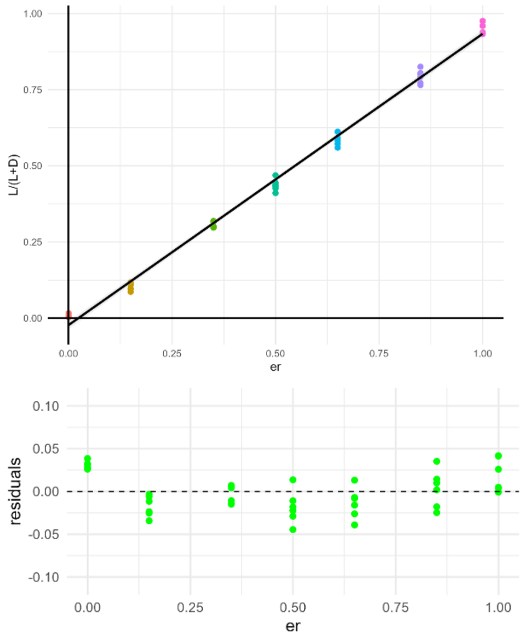	Y = - 0.0015 + 0.8003 X + 0.1563 X^2<br>R^2=0.9971, p-value < $2.2x10^{-16}$<br>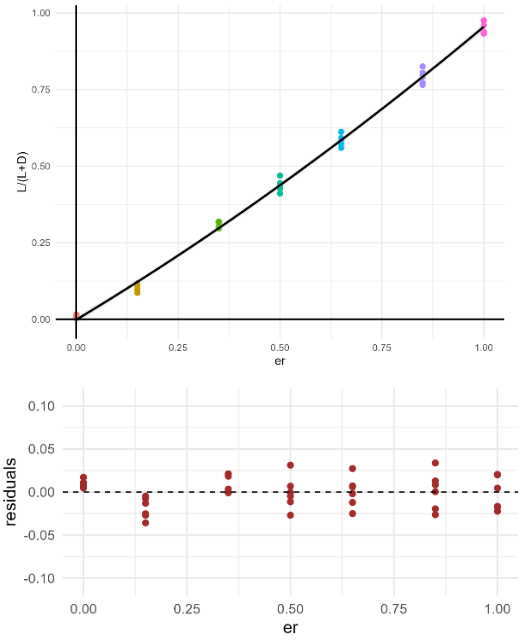
**His**	Y = 0.0244 + 0.8744 X<br>R^2=0.9445, p-value < $2.2x10^{-16}$	Y = 0.1164 + 0.2239 X + 0.6525 X^2<br>R^2=0.9914, p-value < $2.2x10^{-16}$

	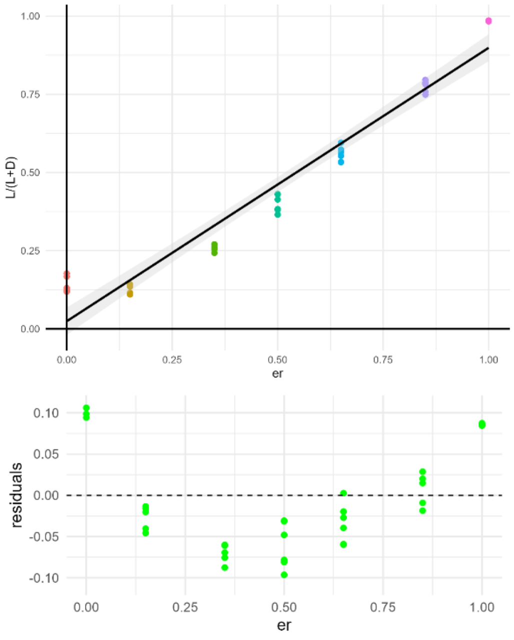	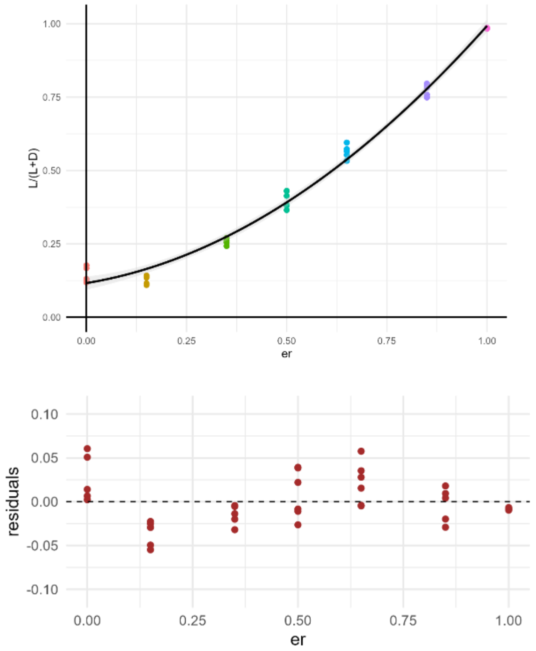
**Met**	Y = 0.0221 + 0.9777 X<br>R^2=0.9853, p-value < 2.2x10^{-16}	Y = 0.0328 + 0.8978 X + 0.0802 X^2<br>R^2=0.9859, p-value < 2.2x10^{-16}
	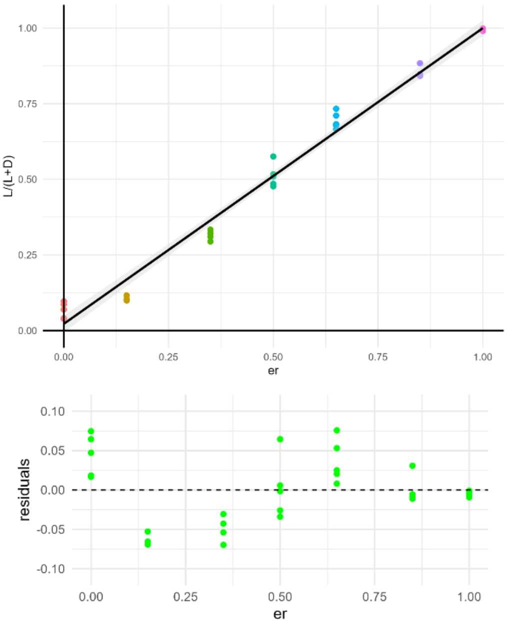	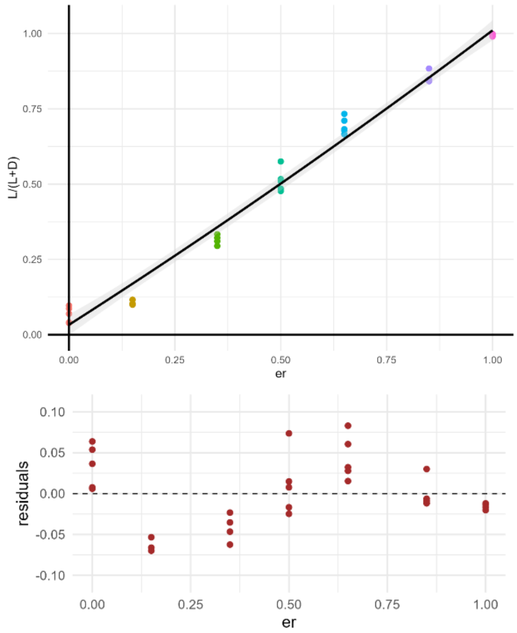
**Pro**	Y = 0.0031 + 0.9788 * X<br>R^2=0.9952, p-value < 2.2x10^{-16}	Y = 0.0187 + 0.8656 * X + 0.1132 * X^2<br>R^2=0.9963, p-value < 2.2x10^{-16}
	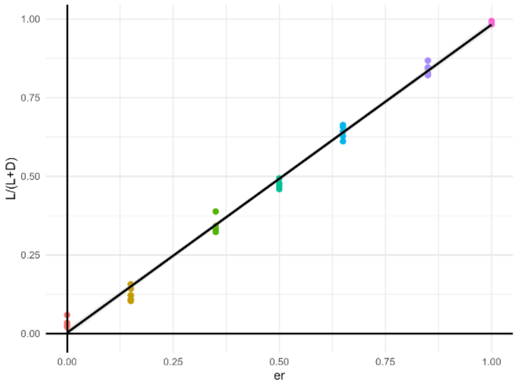	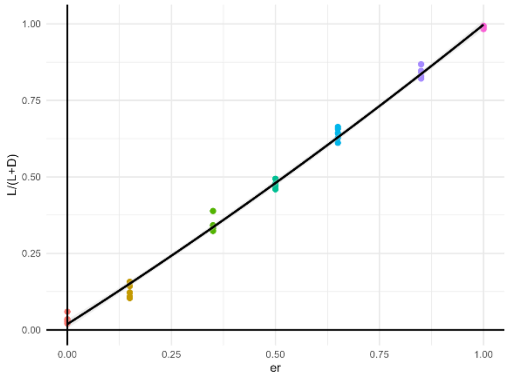

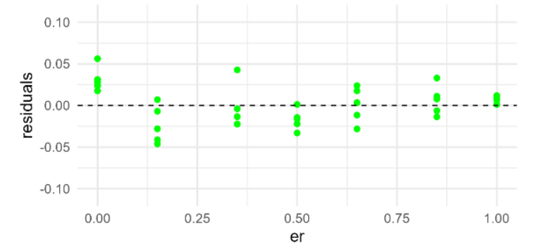

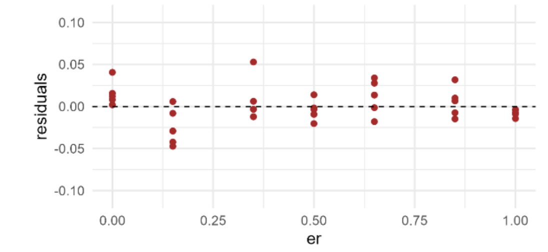

Trp

$Y = -0.0557 + 0.969 X$
R^2=0.9738, p-value < $2.2x10^{-16}$

$Y = 0.0161 + 0.4467 X + 0.5229 X^2$
R^2=0.9978, p-value < $2.2x10^{-16}$

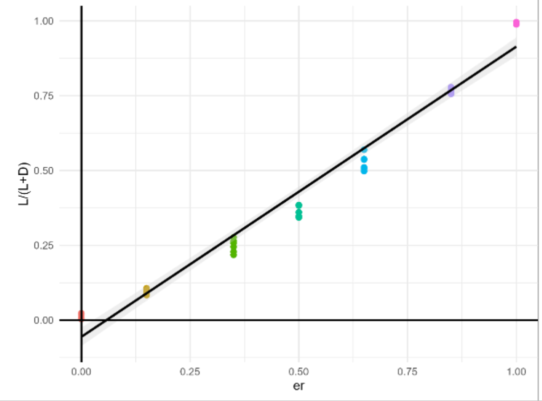

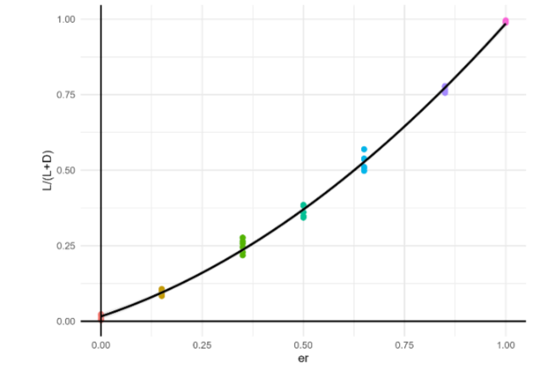

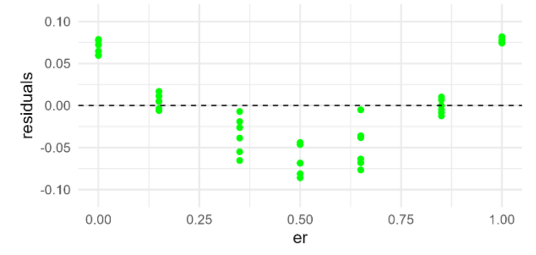

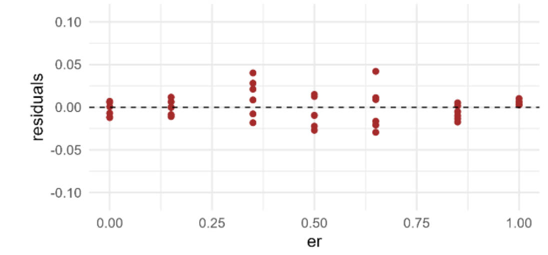

Tyr

$Y = -0.0497 + 0.9635 * X$
R^2=0.976, p-value < $2.2x10^{-16}$

$Y = 0.0199 + 0.456 * X + 0.5075 * X^2$
R^2=0.9989, p-value < $2.2x10^{-16}$

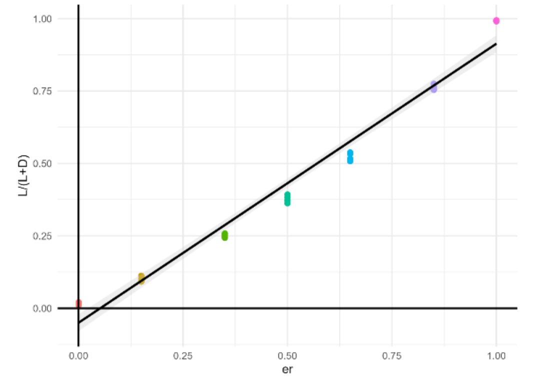

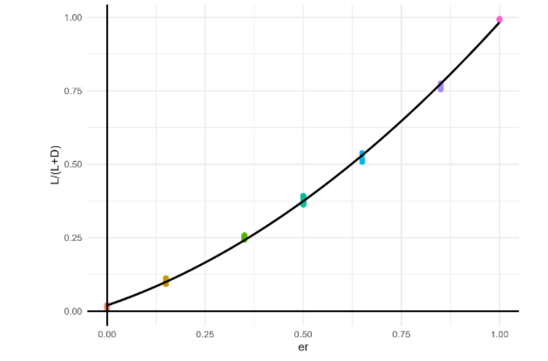

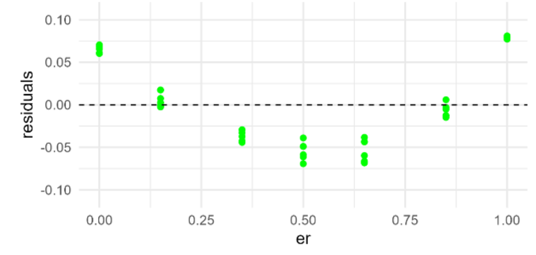

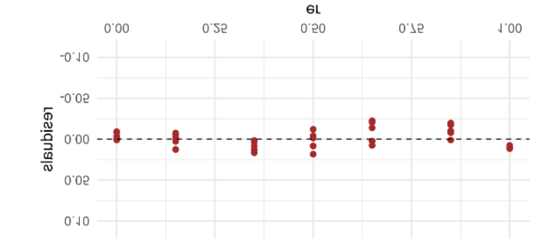

Table S5. Results of regression analysis based on the data from the FIA-SIM²-MS analysis of AA standard solutions at 5 µM concentration. All calibration curves are showed with 95% confidence intervals (not visible for some curves due to their narrow range).

<table>
<tr><th></th><th>Linear model</th><th>Quadratic model</th></tr>
<tr><td>Arg</td><td>$Y = 0.0032 + 0.9762 X$
R^2=0.9995, p-value < $2.2x10^{-16}$</td><td>$Y = 0.0113 + 0.9203 X + 0.0549 X^2$
R^2=0.9998, p-value< $2.2x10^{-16}$</td></tr>
<tr><td></td><td>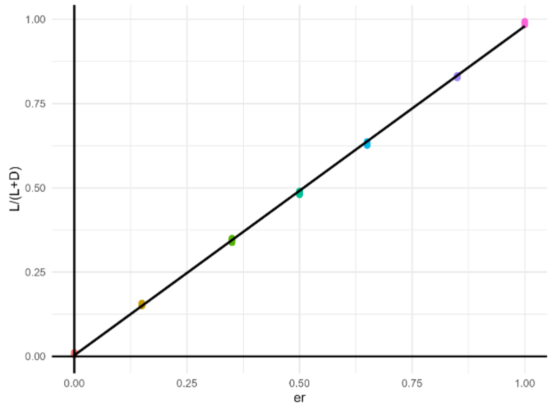
</td><td>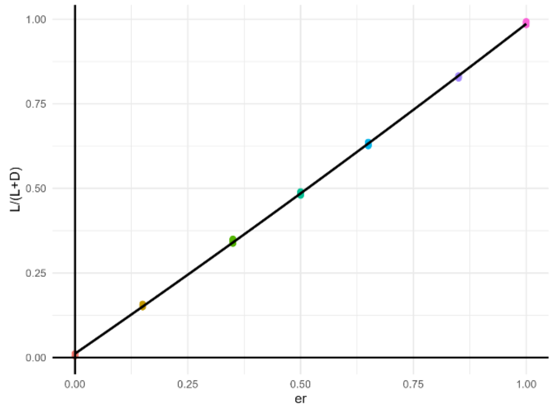
</td></tr>
<tr><td></td><td>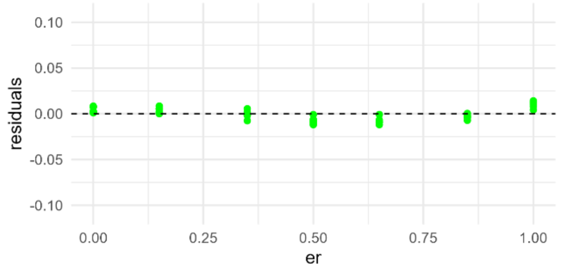
</td><td>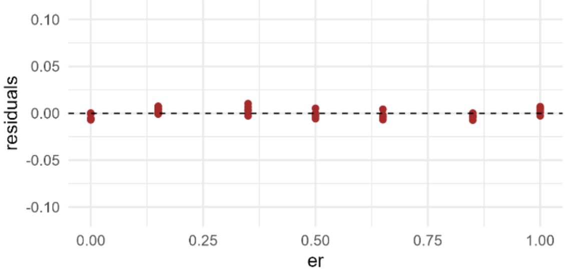
</td></tr>
<tr><td>Gln</td><td>$Y = -0.0211 + 0.9434 X$
R^2=0.9947, p-value < $2.2x10^{-16}$</td><td>$Y = 0.0021 + 0.7835 X + 0.1571 X^2$
R^2=0.9970, p-value < $2.2x10^{-16}$</td></tr>
<tr><td></td><td>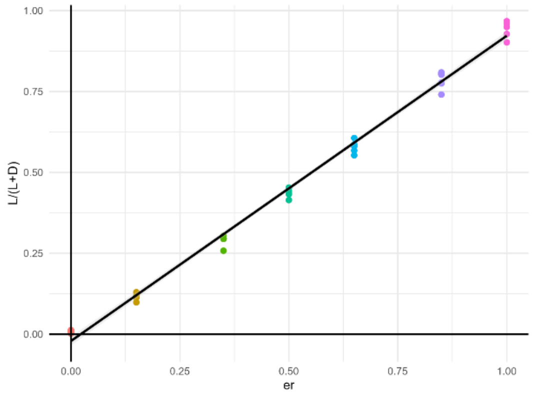
</td><td>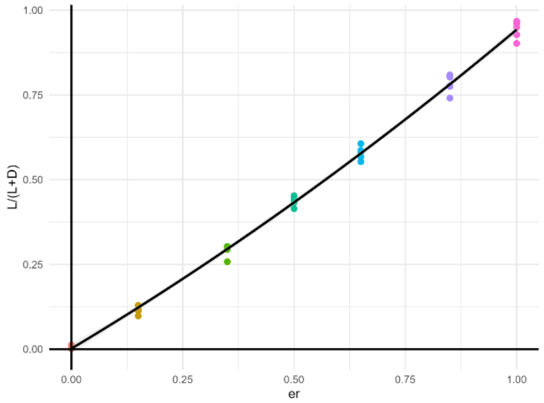
</td></tr>
<tr><td></td><td>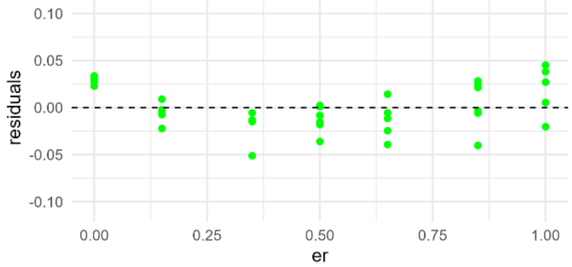
</td><td>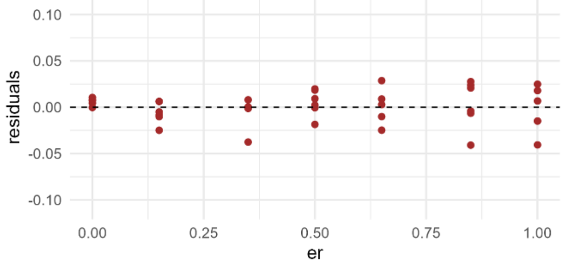
</td></tr>
<tr><td>His</td><td>$Y = - 0.0386 + 0.9908 X$
R^2=0.9853, p-value < $2.2x10^{-16}$</td><td>$Y = 0.0141 + 0.6275 X + 0.3568 X^2$
R^2=0.9959, p-value < $2.2x10^{-16}$</td></tr>
</table>

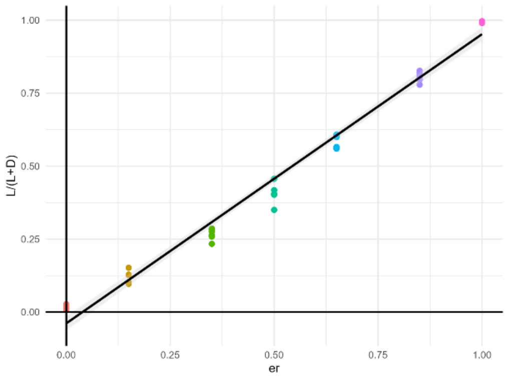

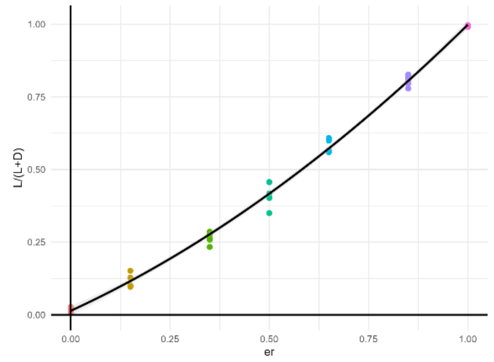

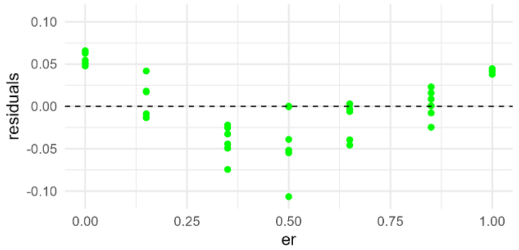

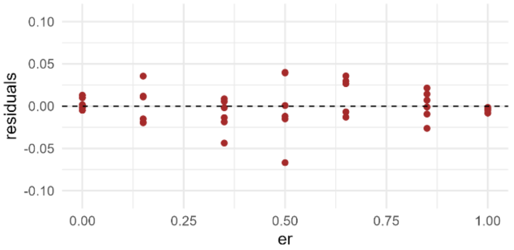

Met

$Y = -0.0403 + 1.003\,X$

$R^2=0.9771$ p-value $< 2.2\times10^{-16}$

$Y = 0.006 + 0.6839\,X + 0.3133\,X^2$

$R^2=0.9851$, p-value $< 2.2\times10^{-16}$

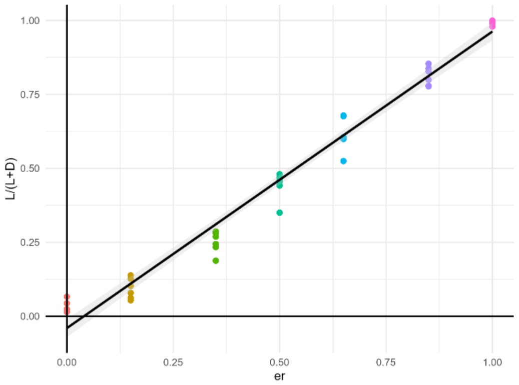

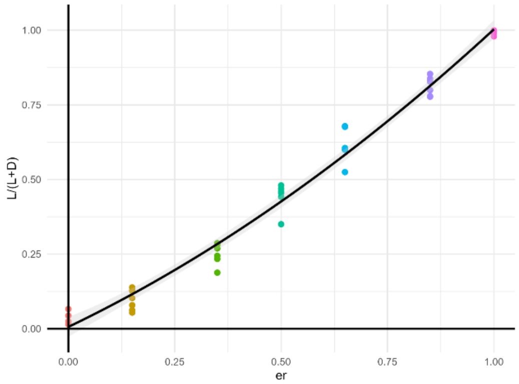

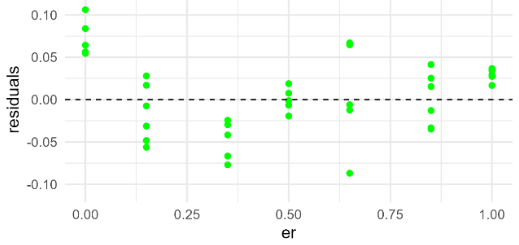

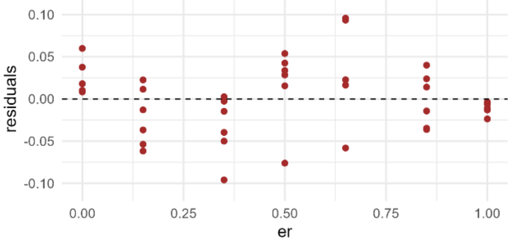

Pro

$Y = -0.0353 + 0.9829\,X$

$R^2=0.9845$, p-value $< 2.2\times10^{-16}$

$Y = 0.0175 + 0.6187\,X + 0.3576\,X^2$

$R^2=0.9954$, p-value $< 2.2\times10^{-16}$

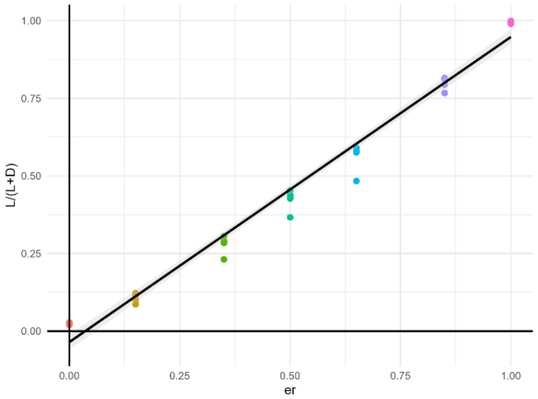

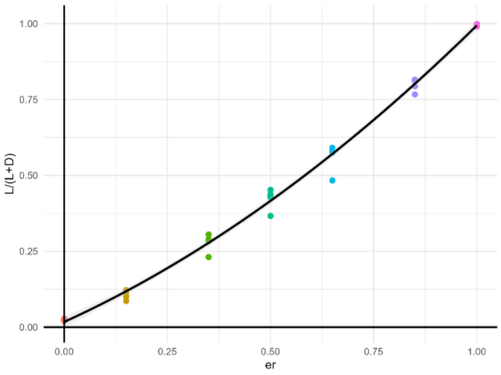

	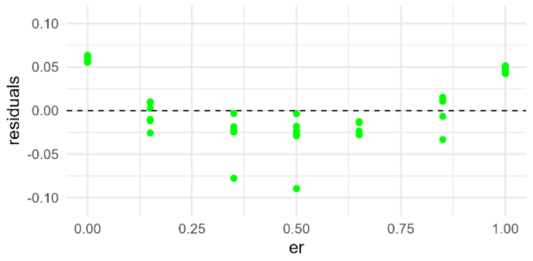	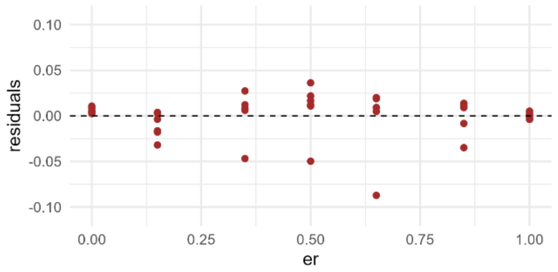
**Trp**	$Y = -0.0790 + 0.984\ X$<br>R^2=0.9674, p-value < 2.2×10^{-16}	$Y = 0.012 + 0.3562\ X + 0.6164\ X^2$<br>$R^2$=0.9991 p-value < 2.2×10^{-16}
	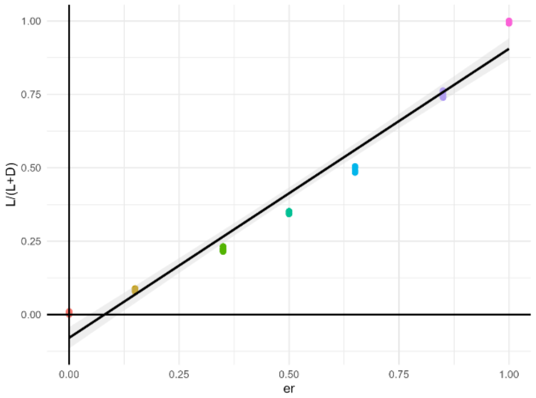	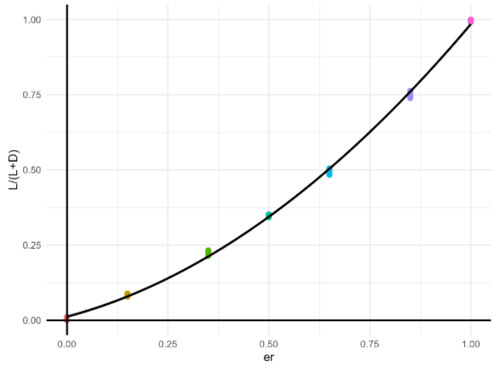
	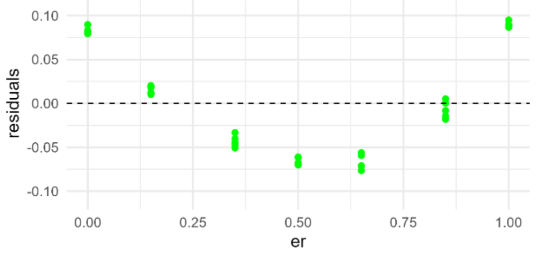	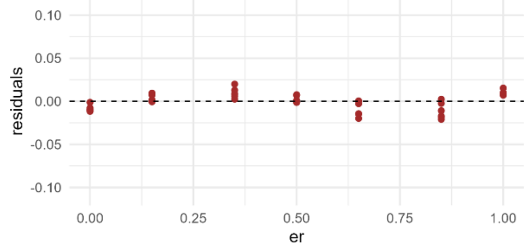
**Tyr**	$Y = -0.0593 + 0.9739\ X$<br>R^2=0.9757, p-value < 2.2×10^{-16}	$Y = 0.0166 + 0.4506\ X + 0.5137\ X^2$<br>$R^2$=0.9984, p-value < 2.2×10^{-16}
	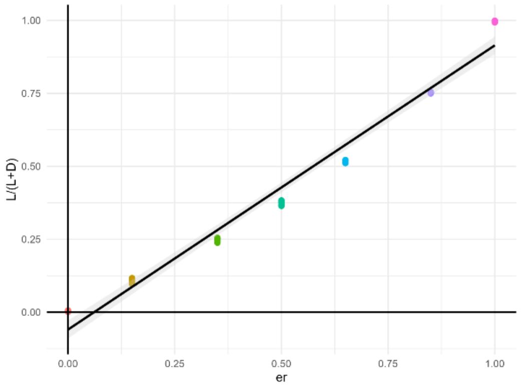	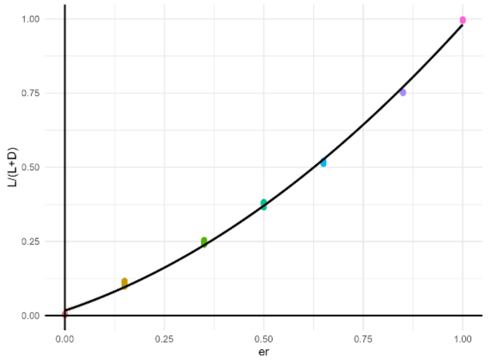
	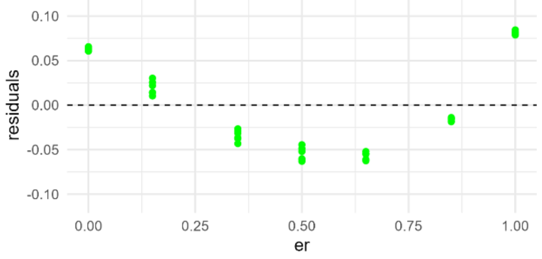	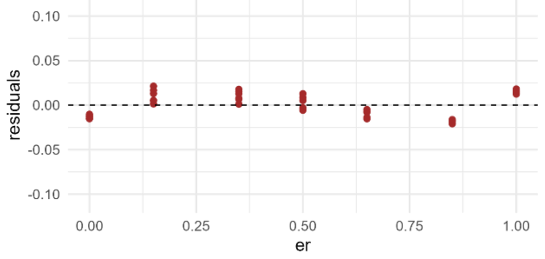

Table S6. Results of the data from the FIA-SIM^2-MS analysis of chiral AA standard solutions at 0.2 µM concentration. All calibration curves are showed with 95% confidence interval.

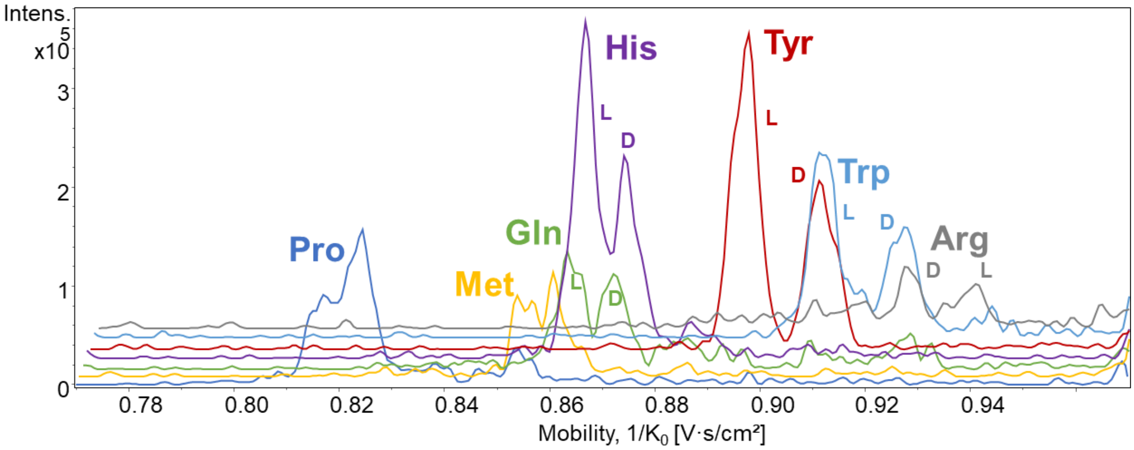

Extracted ion mobility spectra of copper dimer [$^{L+D}$AA+^{L}Phe-2H+CuII]$^+$ ions from SIM^2 analysis of a racemic AA mixture (er=0.5). Each AA (L and D enantiomers) was at a concentration of 0.2 µM.

	**Linear model**	**Quadratic model**
**Arg**	Y = 0.1856 + 0.7074 X<br>R^2=0.9675, p-value=1.346x10^{-15}	Y = 0.1919 + 0.6614 X + 0.046 X^2<br>R^2=0.9678, p-value= 3.696x10^{-14}
**Gln**	Y = 0.0234 + 0.8292 X<br>R^2=0.9717, p-value=3.639 10^{-16}	Y = 0.0581 + 0.5768 X + 0.2524 X^2<br>R^2=0.9793, p-value=7.055 10^{-16}

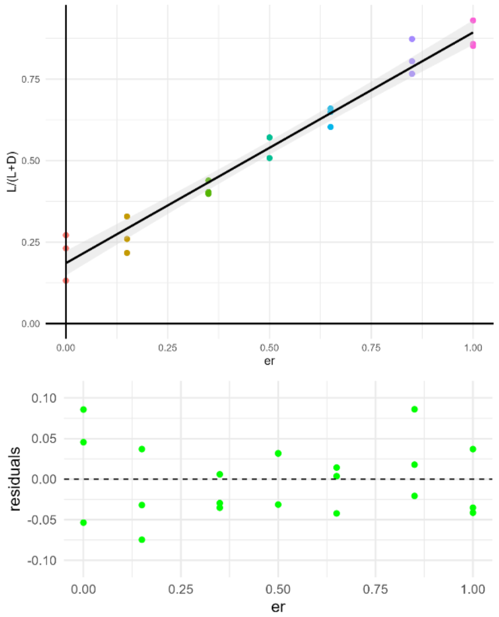

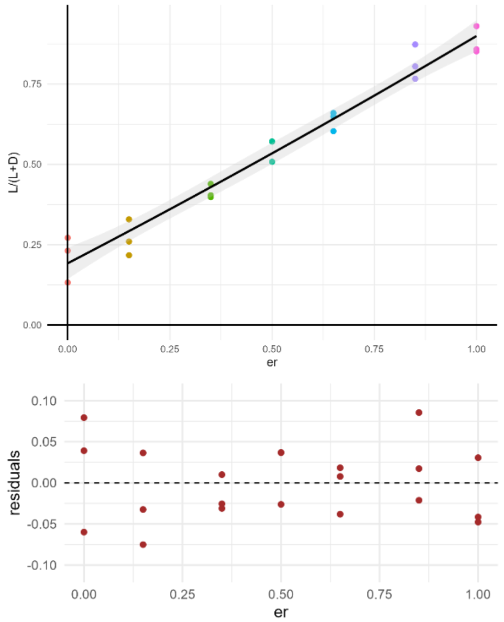

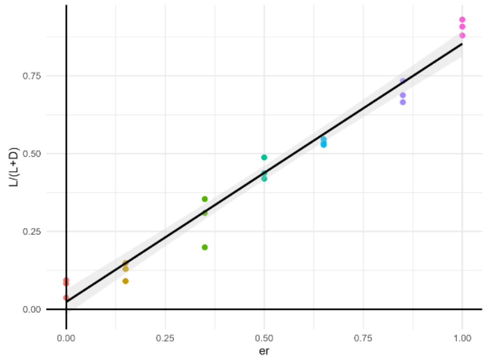

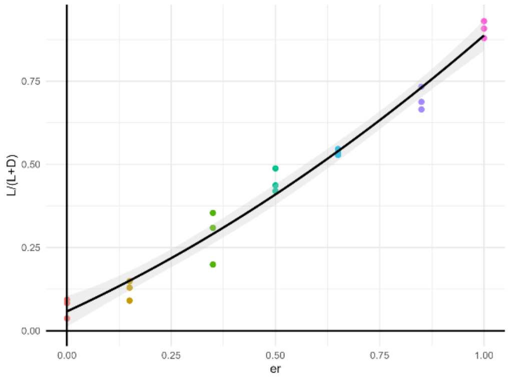

<table>
<tr><td></td><td>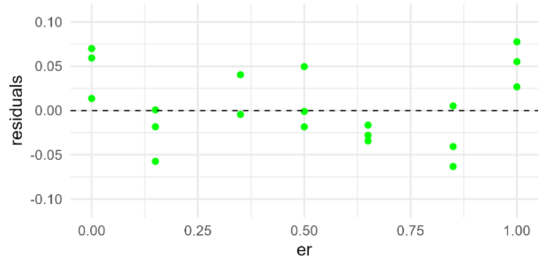
</td><td>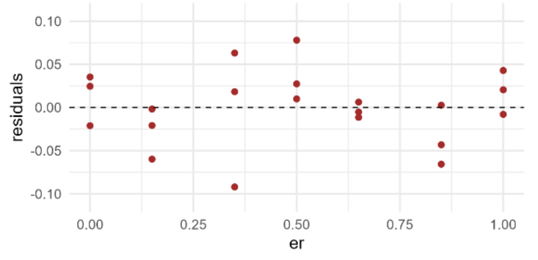
</td></tr>
<tr><td>His</td><td>Y = 0.2138 + 0.5017 X
R^2=0.5296, p-value=0.0002
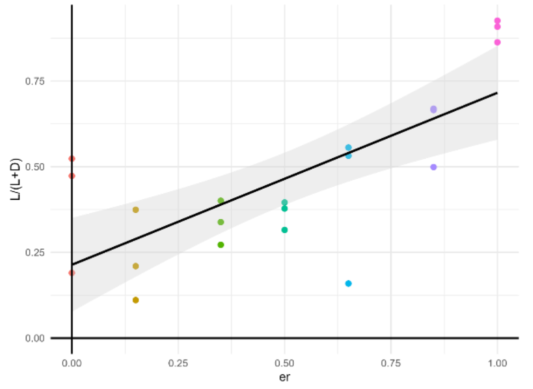

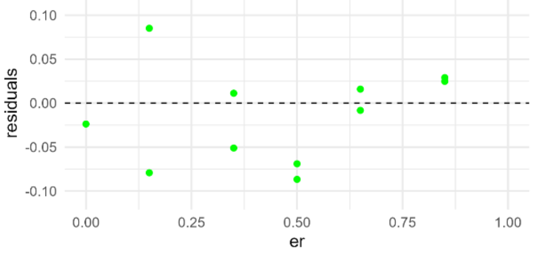
</td><td>Y = 0.3711 - 0.645 * X + 1.1467 X^2
R^2=0.7639, p-value: 2.284 10^{-06}
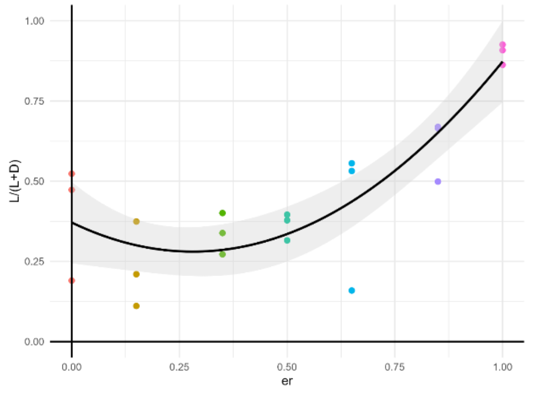

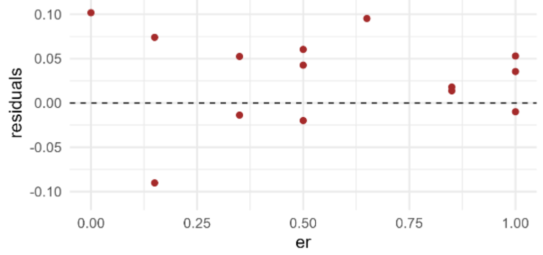
</td></tr>
<tr><td>Trp</td><td>Y = -0.038 + 0.9241 X
R^2=0.9676, p-value: 1.309 10^{-15}
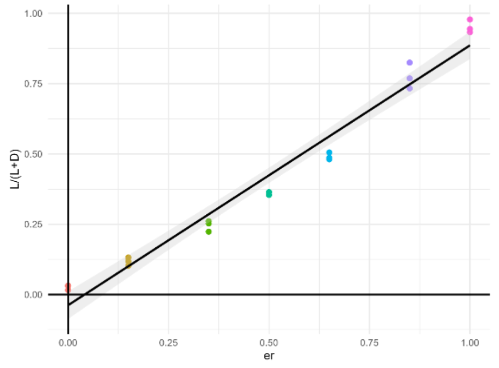

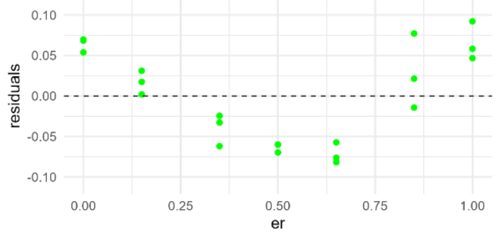
</td><td>Y = 0.0347 + 0.3939 X + 0.5302 X^2
R^2=0.9945, p-value < 2.2x10^{-16}
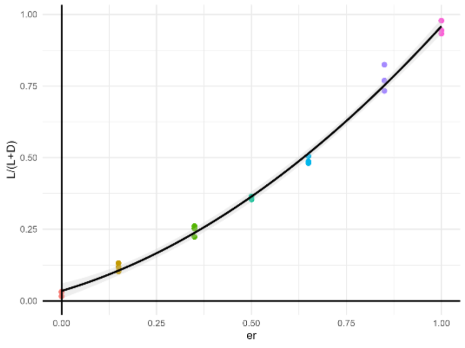

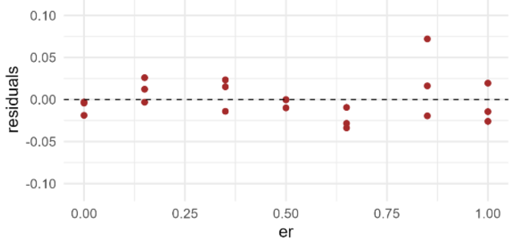
</td></tr>
<tr><td>Tyr</td><td>Y = -0.0387 + 0.932 X
R^2=0.9760, p-value < 2.2x10^{-16}</td><td>Y = 0.0288 + 0.44 X + 0.492 X^2
R^2=0.9991, p-value < 2.2x10^{-16}</td></tr>
</table>

	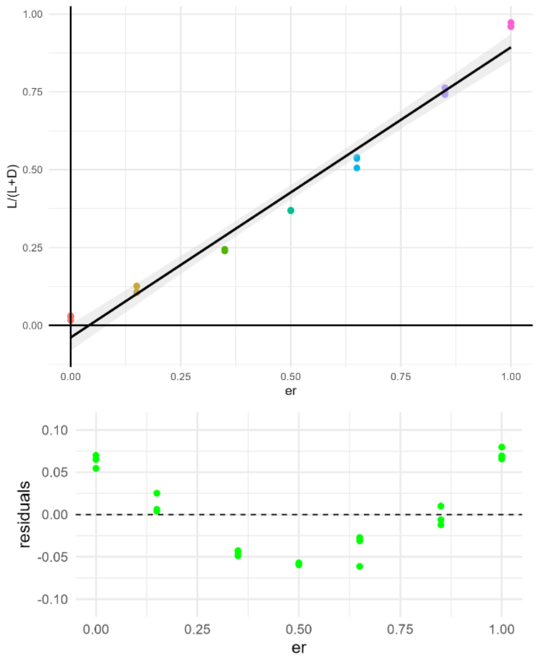L/(L+D)<br>1.00<br>0.75<br>0.50<br>0.25<br>0.00<br>0.00 0.25 0.50 0.75 1.00<br>er<br>residuals<br>0.10<br>0.05<br>0.00<br>-0.05<br>-0.10<br>0.00 0.25 0.50 0.75 1.00<br>er	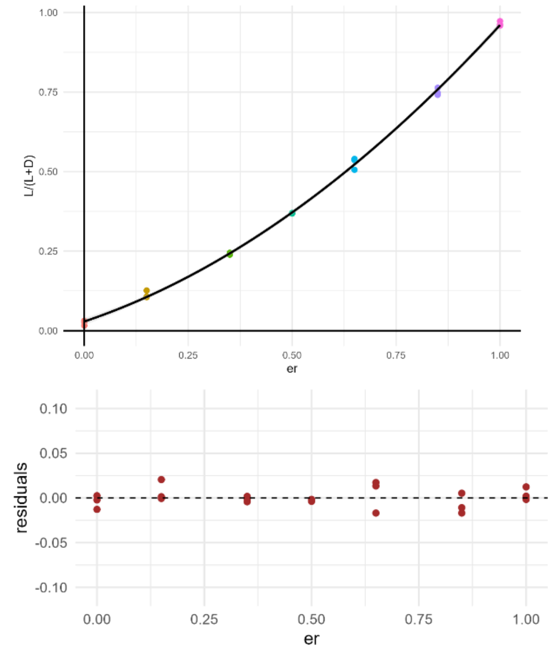L/(L+D)<br>1.00<br>0.75<br>0.50<br>0.25<br>0.00<br>0.00 0.25 0.50 0.75 1.00<br>er<br>residuals<br>0.10<br>0.05<br>0.00<br>-0.05<br>-0.10<br>0.00 0.25 0.50 0.75 1.00<br>er
